# An online paradigm for exploring the self-reference effect

**DOI:** 10.1371/journal.pone.0176611

**Published:** 2017-05-04

**Authors:** Sarah V. Bentley, Katharine H. Greenaway, S. Alexander Haslam

**Affiliations:** The University of Queensland, School of Psychology, Brisbane, Queensland, Australia; Eberhard-Karls-Universitat Tubingen Medizinische Fakultat, GERMANY

## Abstract

People reliably encode information more effectively when it is related in some way to the self—a phenomenon known as the self-reference effect. This effect has been recognized in psychological research for almost 40 years, and its scope as a tool for investigating the self-concept is still expanding. The self-reference effect has been used within a broad range of psychological research, from cultural to neuroscientific, cognitive to clinical. Traditionally, the self-reference effect has been investigated in a laboratory context, which limits its applicability in non-laboratory samples. This paper introduces an online version of the self-referential encoding paradigm that yields reliable effects in an easy-to-administer procedure. Across four studies (total *N* = 658), this new online tool reliably replicated the traditional self-reference effect: in all studies self-referentially encoded words were recalled significantly more than semantically encoded words (*d* = 0.63). Moreover, the effect sizes obtained with this online tool are similar to those obtained in laboratory samples, and are robust to experimental variations in encoding time (Studies 1 and 2) and recall procedure (Studies 3 and 4), and persist independent of primacy and recency effects (all studies).

## Introduction

What is *the self*? The answer to this question is intimately tied to the tools available to study it, and thus knowledge gleaned about the self often represents the zeitgeist associated with different eras of psychological research. However, one particular methodological approach to studying the self—the self-reference effect—has stood the test of time, and remains as relevant today as when it first emerged in the 1970s [1–4]. Looking back, Craik and Tulving’s [5] ground-breaking work on how memory is influenced by encoding style was the precursor to the development of what became known as *self-referential encoding*. Reliably demonstrated across over 100 studies [6], this effect shows that information leaves a deeper and more robust memory trace when it is encoded with reference to the self.

Craik and Tulving [5] conducted a series of rigorous studies examining the effects of different ‘depths’ of encoding. Their results reliably showed that semantic encoding produced the most robust and long-standing memory trace, and that this result was not correlated with the length of time spent encoding. Their studies supported Craik and Lockhart’s [7] Depth of Processing Model, which is still highly influential today. It was not long, however, before Rogers and colleagues [8] began to investigate a different, and even more powerful, type of encoding. These authors compared structural, phonetic, and semantic encoding with *self-referential* encoding—such as thinking about whether a word described the self. The findings showed that self-referential encoding led to significantly deeper memory traces than all other encoding types [8]. This has since been confirmed in hundreds of studies. Indeed, Symons and Johnson (1997) conducted a large-scale meta-analysis of 129 published studies using the self-reference paradigm in an attempt to assess the strength and consistency of the effect, and concluded that self-referential encoding is the most effective level of encoding for promoting memory, and that this effect is robust to a variety of experimental variations [6]. Despite debate continuing as to what lies behind the self-reference effect (for instance the role of elaboration or schematic organization), self-referential encoding remains a robust phenomenon able to reveal much about the way information is processed.

The standard experimental self-reference paradigm occurs in a laboratory context. Encoded words are generally single adjectives (e.g., courageous), presented to participants orally by an experimenter, on pieces of card, or on monitors. Participants are given paper-based answer booklets with the encoding questions listed (e.g., “Does this word describe yourself?”), and a space to answer either ‘Yes’ or ‘No’. Questions are randomly sequenced according to whether they represent self-referential encoding, semantic encoding, phonetic encoding, or other levels of encoding under investigation. After participants have completed the sequence of words and associated questions (usually upwards of 30 words in a sequence), they are then asked to perform a filler task—something to avoid non-conscious rehearsal effects. Once completed, they are then given a surprise memory test. This incidental recall task is normally presented as a free-recall activity in which participants are given two or three minutes to recall as many words as possible from the encoding task. The dependent variable is the number of words successfully recalled.

The self-reference effect has informed research in a number of psychological areas. Research on the self-concept and self-attention has benefitted from this paradigm [9], as well as appraisal research on the influence of the self in perception and interpretation [10]. Klein and Loftus [11] used the self-reference effect to better understand autobiographical memory, and more recently the self-reference effect has been used to advance understanding of implicit and explicit cognition [12]. Furthermore, the self-reference effect has been used to progress potential memory enhancement strategies for individuals with neurological damage [4] and has stimulated a rich line of enquiry in the emerging field of neuroscience, with the behavioral data obtained using the traditional self-referential paradigm enriching investigations into the neural underpinnings of the self [3, 13, 14]. Finally, the self-reference effect has also proved to be a highly useful tool in the analysis of different cognitive styles within a range of cultural groups [15–17].

The self-reference effect has also played a key role when trying to understand clinical populations, most notably in exploring the self-concept of individuals on the autistic spectrum [18, 19], those diagnosed with schizophrenia [20], and individuals with differing levels of depression [2, 21]. In general, research shows the self-reference effect is less present, or negatively biased, in these samples. For instance, clinical research with depressed individuals demonstrates tendencies to have a significant negative bias when encoding adjectives—that is, these respondents endorse and recall more negative words than positive words [21, 22]. These findings are reinforced by research using neurological measures, with results supporting this more maladaptive self-view [2]. This pattern of findings is also evident in research on personality disorders, such as borderline personality disorder [23]. Furthermore, research suggests that the self-reference effect is less evident in individuals on the autism spectrum [18], and absent in individuals with schizophrenia [20]. Non-clinical samples, on the other hand, tend to show patterns of self-referential encoding that reflect self-serving attribution biases [24, 25].

Recently, studies have investigated the test-retest reliability of the self-reference effect, and have confirmed its robustness, particularly when examining behavioural and neural correlates over time [26, 27]. This broad literature demonstrates the influence of the self-referential effect in progressing a core understanding of self-structure and self-function in both healthy and clinical populations.

These various lines of research speak to the fact that the self-reference paradigm holds a cherished place within the pantheon of research tools available for studying the self. Moreover, it seems likely that it will continue to hold sway as new ways of investigating the self are uncovered and explored. However, its operational delivery in a traditional laboratory context means that the empirical power of the self-reference paradigm remains limited. Across all of the self-referential encoding studies analyzed by Symons and Johnson [6], the average sample size per study was 39, and 82% of all studies were conducted using college undergraduates as participants. Some experimenters have attempted to expand the potential participant audience by projecting words onto a large screen in order to test multiple participants at the same time [28]. However, Symons and Johnson’s (1997) meta-analysis revealed that this procedure of testing more participants in the one session through the use of projections resulted in significant decreases in self-referential encoding in some procedures [6]. With current debate keenly focused on the implications of low statistical power for both Type I and Type II errors [29], as well as low replication rates [30, 31], the self-reference paradigm would benefit from moving beyond the confines of the laboratory to online settings in which statistical power can be maximized.

The importance of progressing scientific understanding of the self and its implications for psychological functioning highlights the need for an updated methodology to study this seminal effect. Accordingly, we believe that researchers would benefit from a new version of the self-referential paradigm—one that can be quickly, easily and inexpensively administered to a large and varied sample of participants. Not only will this allow for sampling beyond the confines of the undergraduate student population, but it will also allow researchers to conduct studies with large between-groups samples. Such developments could also be a basis for efforts to better understand individual differences in self-referential encoding outcomes, as well as for more experimental analyses of contextual influences on self-referential encoding.

In this paper we respond to this demand for power and accessibility by seeking to develop an online version of the self-referential paradigm. More specifically, we present the results of four experiments that test the reliability of a new online self-referential encoding tool using a participant data pool provided through Amazon’s Mechanical Turk (MTurk). Studies 1 and 2 describe preliminary formulations of the paradigm, conducted with increasingly large participant data sets. Two further studies test the reliability of the online self-reference effect across experimental variations used in traditional laboratory-based studies—comparing a recall task with a recognition task (Study 3), and an informed recall task with an incidental recall task (Study 4). These four studies allow us to test the validity and reliability of the self-referential effect using a screen-only delivery method, and to investigate this delivery method using a crowd-sourcing platform.

## Study 1

Study 1 presents the initial test of our new online self-referential encoding paradigm, which was created using Qualtrics software, and distributed on the MTurk platform. Symons and Johnson’s meta-analysis (1997) revealed a high level of variability regarding the mode of presentation, whether using a projector, a tachistoscope, index cards or booklets. The purpose of this first study was therefore to test for a self-reference effect within this entirely online experimental context. We hypothesized that the study would reveal a typical self-reference effect such that participants recalled significantly more self-referentially encoded words than semantically or structurally encoded words.

### Method

#### Ethics statement

For this and all subsequent studies, ethical clearance was obtained from the Behavior and Social Sciences Ethical Review Committee (BSSERC) at the University of Queensland. As in all studies reported below, before completing the online experiment, participants were informed about the aims of the study and provided consent to participate, and after completing the study participants were fully debriefed.

#### Participants and design

Participants were 103 MTurk Workers (44% women, *M*_age_ = 36.71, *SD*_*age*_ = 12.85, range 19–71). MTurk participants were paid USD$0.50 to take part in a 3 (encoding level: self-referential, semantic, structural) 3 X 2 (filler task: present, absent) mixed design. Presence (vs. absence) of the filler task was a between-subjects variable and encoding type was a within-subjects variable. The dependent variable was the proportion of correct words recalled as a function of encoding level. Thus, our main dependent variable of interest was the number of self-referentially encoded words divided by the total number of correct words recalled by each participant. This proportion was also calculated for semantic and structurally encoded words. Correct words were defined as words that were an orthographically approximate match of the word [5] used at the encoding phase (e.g., deceit and deciet).

An a priori power analysis was completed based on the effect size of *d* = 0.65 for self-referential encoding compared to semantic encoding as reported in Symons and Johnson’s large-scale meta-analysis (1997). The power analysis indicated that with power at 95% and an alpha of .05, a sample size of 33 would be sufficient to detect an effect of self-referential encoding. As this study was a first test of the online effect, which might be expected to be smaller than in laboratory samples, and as we also investigated the presence and absence of a filler task between encoding and recall, we made an a priori decision to recruit a conservative sample size of 100 participants.

#### Procedure and materials

After providing consent, participants were presented with a brief explanation of the encoding task (described as a ‘word processing task’). They were then provided with an example question and answer, and went on to complete the encoding task, which involved answering questions about 30 words (see ‘Encoding paradigm’ below). Participants were then randomly allocated to the filler or no filler task condition and completed the ‘surprise’ incidental recall phase, in which they were asked to recall as many of the words presented during the encoding task as possible. They were given 120 seconds to complete the recall task, and instructed to recall as many words as possible in any order. They were then asked for some basic demographic information. Finally, participants were debriefed and paid.

#### Encoding paradigm

During the encoding phase, participants were presented with an encoding question, a word, and the answer choices (‘Yes’ or ‘No’) on the same screen. Each screen was also subtitled with the question number and the total number of questions (e.g., 5 of 30), so that participants could monitor their progress as they worked through the series of questions. Each question was presented centrally at the top of the screen in a 12-point sans-serif font (Arial). The word to which the question referred was presented centrally under the question in a 72-point lower-case bold sans serif font (Arial). Of the 30 words, two had a substantially increased character count, and these two words were displayed at a reduced font size of 48-point. All other words were displayed in a larger font to maximize visibility on electronic devices. See S2 Appendix for details of the word lists.

Under the word, two multiple choice answers were presented: ‘Yes’ or ‘No’. These words were presented in a 10-point sans-serif font (see S3 Appendix). Once participants selected their answer, they were automatically forwarded to the next question screen. In this study there was no time constraint set on the presentation of the screens, so participants could click through the task as quickly or as slowly as they wished.

Participants completed encoding questions related to 30 adjectives. The adjectives were chosen from Anderson’s ‘Likeableness’ ratings of 555 personality-trait words’ [32], which has been used as a word source for a large number of experimental self-referential paradigms over the course of the last 40 years. Words were selected to represent a diverse range of personality traits (see S2 Appendix). The choice of words was selected for range length (six words of one syllable, seven words of two syllables, nine words of three, and eight words of four syllables), and were matched on word length and valence: 15 words were positive and 15 words were negative. The equal ratio of positive to negative words was maintained for each encoding level. As such, half of the self-referential words were positively valenced and half were negatively valenced, and so forth for semantic and structural words. For the 30 words, each participant was asked 10 self-referential questions (“Does the following word describe yourself?”), 10 semantic questions (“Does the following word mean [e.g., courageous]?”), and 10 structural questions (“Is the following word written in upper case?”). In the case of the semantic questions, an equal number of synonyms and antonyms were chosen from Roget’s Thesaurus online [33]. Furthermore, the structural and semantic questions were counterbalanced for answer choice, such that 5 were chosen to lead to a positive “Yes” answer (e.g. “Is the following word [TACTFUL] written in upper case?”), and 5 were chosen to lead to a negative “No” answer (e.g. “Does the following word [cowardly] mean ‘bold’?”).

In order to remove variation due to potential word/question association and word presentation order, six different pseudo-randomized versions of the encoding lists were created and participants were randomly selected to receive one of these six lists. For each list the same words were used, but were associated with a different question (encoding type and/or affirmative/negative answer), and in each list the words were presented in alternative pseudo-randomized orders. Analyses revealed no significant effect of list; therefore this factor was collapsed across all studies.

#### Filler task

The filler task comprised 5 math questions, each with three possible multiple choice answers. These questions were designed to be moderately difficult (e.g., what is the sum of 112 + 49?).

#### Attention check and demographics

As recommend by Meade and Craig [34] we embedded one attention check in the demographics presented at the end of the study (“For this question, please just click the option ‘Very much’”). One participant failed the attention check. Excluding this participant from analyses did not substantively change the results, but their data were nevertheless excluded from further analysis. Participants also indicated their age, gender and level of education.

### Results

Data were analyzed using a 3 X 2 mixed-design ANOVA with a within-subjects factor of encoding level (self-referential, semantic, structural) and a between-subjects factor of filler task (filler task, no filler task). The dependent variable was the proportion of words correctly recalled at each level of encoding. Many investigations of the self-reference effect guard against the possible memory effects of primacy and recency by discounting words recalled by participants that appeared in the first or last three positions in the encoding list [8, 35, 36]. We therefore performed all main analyses using both the full (i.e., liberal) data set and the truncated, conservative data set (which excluded recalled words that appeared in the first or last three positions in the encoding list). The means and standard deviations for the main effect of encoding in all studies are displayed in Table 1.

**Table 1:** Mean proportion of correct responses as a function of encoding condition and data set (liberal vs. conservative; standard deviations in parentheses).

Study and data set		SRE	SEM	STR
**Study 1**	Conservative data	44.48% (29.79)	39.59% (30.31)	10.04% (16.87)
	Liberal data	47.41% (27.19)	36.29% (26.01)	13.36% (17.79)
**Study 2**	Conservative data	50.35% (37.37)	26.13% (30.95)	7.29% (18.22)
	Liberal data	49.52% (30.16)	24.67% (26.65)	14.12% (20.04)
**Study 3 Recall**	Conservative data	51.73% (34.45)	30.49% (30.69)	10.78% (18.71)
	Liberal data	50.12% (27.47)	28.92% (23.61)	10.78% (18.71)
**Study 3 Recog**.	Conservative data	41.27% (9.07)	40.77% (9.77)	15.96% (18.34)
	Liberal data	43.50% (8.19)	37.35% (8.05)	19.15% (10.37)
**Study 4**	Conservative data	43.83% (33.22)	29.94% (28.56)	16.90% (21.63)
	Liberal data	44.49% (27.05)	27.11% (23.29)	20.65% (18.71)

*Notes*: Conservative data: excludes any words recalled that occurred in the first three positions or last three positions of the encoding list. Liberal data: includes all words recalled including those that occurred in the first three positions or last three positions of the encoding list.

SRE = self-referential encoding; SEM = semantic encoding; STR = structural encoding.

#### Conservative analyses: Primacy and recency words excluded

Mauchley’s test indicated that the assumption of sphericity was violated (*W* = 0.73, *p* < .001), therefore degrees of freedom were corrected using Huynh-Feldt estimates of sphericity. There was a non-significant main effect of filler task, *F*(1,101) = 0.02, *p* = .880, η_p_^2^ < .001, but a significant main effect of encoding *F*(2,202) = 35.79, *p* < .001, η_p_^2^ = .241. We used the *lme4* package [37] in R [38] to perform a linear mixed-effects analysis. In contrast to a more traditional approach with data aggregation and repeated-measures ANOVA analysis, *lme4* controls for the variance associated with random factors without resorting to data aggregation (for a discussion see [39]; [40]). In this model we included a fixed effect of encoding level and a random intercept for participants. Participants recalled no more self-referentially encoded words (*M* = 44.05%, *SD* = 29.27) than semantically encoded words (*M* = 39.21%, *SD* = 30.41), *β* = -4.84, SE = 3.67, *p* = .385, but recalled more self-referentially encoded words than structurally encoded words (*M* = 9.95%, *SD* = 16.82), *β* = -34.11, SE = 3.67, *p* < .001. Participants recalled significantly more semantically encoded words than structurally encoded words, *β* = -29.26, SE = 3.67, *p* < .001. The interaction was non-significant, *F*(2,202) = 0.72, *p* = .487, η_p_^2^ = .006.

#### Liberal analyses: Primacy and recency words included

The effects were similar using the liberal data that included primacy and recency words. The main effect of filler task remained non-significant, *F*(1,101) = 0.03, *p* = .860, η_p_^2^ < .01, and the main effect of encoding remained significant, *F*(2,202) = 36.66, *p* < .001, η_p_^2^ = .25. Planned contrasts revealed a significant difference between self-referentially encoded and semantically encoded words, *β* = -11.01, SE = 3.35, *p* = .003, and self-referentially encoded and structurally encoded words, *β* = -33.72, SE = 3.35, *p* < .001. The interaction between filler task and encoding was again non-significant, *F*(2,202) = 0.07, *p* = .930 η_p_^2^ < .001.

Using the liberal data set, the potential impact of age, gender and education levels were analyzed using a mixed multilevel model. Results revealed no main or interactive effects for age (*p*s > .396), gender (*p*s > .404), or education (*p*s > .621). We also checked whether the effect of encoding differed for positively and negatively valenced words. A a paired *t*-test revealed that positive words were recalled significantly more than negative words, *p* < .001, 95% CI [18.21, 35.50], thus indicating a general bias towards positive words [41]. However, further analyses revealed that the main effect of encoding remained significant for both positively valenced recalled words, *F*(1,204) = 29.87, *p* < .001, η_p_^2^ = .20, and negatively valenced recalled words, *F*(1,204) = 6.50, *p* < .001, η_p_^2^ = .05. The simple comparisons mirrored the main effect comparisons. Such findings are consistent with the standard patterns seen in generalized non-clinical populations [18, 20]. Means and standard deviations for these valence-based supplementary analyses are presented in Table 2.

**Table 2:** Proportion of correct responses as a function of encoding condition and valence, using liberal data sets.

	SRE		SEM		STR	
	Positive	Negative	Positive	Negative	Positive	Negative
**Study 1**	32.77%	16.60%	23.53%	13.03%	6.30%	7.77%
**Study 2**	32.33%	23.83%	16.67%	11.50%	8.83%	6.84%
**Study 3**	30.46%	21.64%	13.43%	16.83%	9.62%	8.02%
**Study 4**	28.62%	19.81%	14.92%	13.61%	12.74%	10.30%

*Note*: Positive = positively valenced words; Negative = negatively valenced words; SRE = self-referential encoding; SEM = semantic encoding; STR = structural encoding

### Discussion

Study 1 confirmed that our novel online encoding paradigm was capable of producing the standard self-reference effect. As hypothesized, participants recalled more self-referentially encoded words than semantically encoded words and structurally encoded words when including primacy and recency words. All differences were in the hypothesized direction and all were significant, with the exception of differences between self-referential encoding and semantic encoding in the conservative data. This pattern is representative of the standard results obtained when comparing self-referential encoding with semantic encoding and structural encoding in more traditional laboratory contexts [6].

Neither the presence of a filler task, nor the removal of recalled words that featured in the first three or last three positions in the encoding list had any substantive impact on general trend of results. The lack of significant difference between self-referential encoding and semantic encoding within the conservative data may be explained by the speed with which participants encoded the words in this study. As there was no time constraint, participants completed the questions as quickly as possible. As we report below, this situation was investigated and resolved in Studies 2, 3 and 4.

Looking to the question of valence, subsequent analyses of the liberal data revealed that participants recalled significantly more positively valenced words than negatively valenced words, which is consistent with research demonstrating a bias in normal populations towards remembering positive information over negative information [22, 42–44].

## Study 2

Study 1 demonstrated that our novel online paradigm successfully produced the standard self-reference effect. However, a significant difference between self-referentially encoded words and semantically encoded words was seen only in the liberal data. A possible reason for this weakening of the self-reference effect was the speed with which participants encoded the information. In Study 1 the design of the online delivery allowed participants to determine the presentation speed of the encoding task—that is, words appeared as soon as participants had selected their answer. On inspection, participants spent an average of two seconds encoding each word. In Study 2 we slowed the encoding phase of the study to mimic the traditional experimenter presentation speed in a laboratory context: approximately 5 seconds per word. As in Study 1, we hypothesized that participants would recall significantly more self-referentially encoded words than semantically or structurally encoded words.

### Method

#### Participants and design

Participants were 150 MTurk Workers (49% women, *M*_age_ = 35.89, *SD*_age_ = 12.40, range 19–70) who were paid USD$0.50 to take part in a one-way repeated measures design (encoding level: self-referential, semantic, structural). Participants who completed Study 1 were excluded from taking part in the study. The dependent variable was the proportion of correct words recalled as a function of encoding level.

#### Procedure and materials

The procedure was identical to Study 1 with the exception that presentation of the encoding task was slowed. This was achieved by breaking down the presentation of each question and its associated word into a timed and standardized sequence of screen displays. For each set of questions, words, and answers, the following sequential pattern was adopted: first, an encoding question (e.g., “Would you use the following word to describe yourself?”) was displayed for 2 seconds; second, the word to be encoded appeared underneath the question; finally, the question and word were displayed together for a further 3 seconds before the answer options ‘Yes’ or ‘No’ appeared below. The transition to the next screen was dependent on the speed with which participants selected their answer. Once all 30 words were encoded, participants then completed the same filler task used in Study 1.

Participants answered encoding questions related to the same 30 adjectives from Study 1. However, there were two particular words that stood out in Study 1 as not being recalled regardless of encoding level. These two words were ‘insincere’ and ‘absent-minded’. Accordingly, these words were substituted in Study 2 for ‘immature’ and ‘discourteous’, respectively, which were considered to be more contemporary and typical within a North American context (see S2 Appendix).

#### Attention check and demographics

We again embedded one attention check in the demographics presented at the end of the study (“For this question, please just click the option ‘Very much’”). Two participants failed the attention check. Excluding these two participants from analyses did not substantively change the results, but their data were nevertheless excluded from further analysis. Participants also indicated their age, gender and level of education.

### Results

The means and standard deviations for the main effect of encoding in all studies are displayed in Table 1.

#### Conservative analyses: Primacy and recency words excluded

Data were analyzed using a one-way repeated measures ANOVA. The dependent variable was the proportion of words correctly recalled at each level of encoding. Mauchley’s test indicated that the assumption of sphericity was violated (*W* = 0.78, *p* < .001), therefore degrees of freedom were corrected using Huynh-Feldt estimates of sphericity. Analysis revealed a significant main effect of encoding type, *F*(2,300) = 63.12, *p* < .001, η_p_^2^ = .26. A linear mixed effects analysis revealed that participants correctly recalled significantly more self-referentially encoded words (*M* = 50.35%, *SD* = 37.37) than semantically encoded words (*M* = 26.13%, *SD* = 30.95), *β* = -11.01, SE = 3.35, *p* < .003, and structurally encoded words (*M* = 7.29%, *SD* = 18.22), *β* = -33.72, SE = 3.35, *p* < .001. Participants also recalled significantly more semantically encoded words than structurally encoded words, *β* = -22.71, SE = 3.35, *p* < .001,

#### Liberal analyses: Primacy and recency words included

The effects were similar using the liberal data. The main effect of encoding remained significant, *F*(2,300) = 60.09, *p* < .001, η_p_^2^ = .25, as did the specific comparisons between self-referentially encoded words and semantically encoded words, *β* = -24.86, SE = 2.95, *p* < .001, and self-referentially encoded and structurally encoded words, *β* = -35.40, SE = 2.95, *p* < .001.

Using the liberal data, the potential impact of age, gender and education levels were analyzed using a mixed multilevel model. Results revealed no main or interactive effects for age (*p*s > .413), gender (*p*s > .319), or education (*p*s > .143). We also checked whether the effect of encoding differed for positively and negatively valenced words. Although a paired *t*-test revealed that positive words were recalled significantly more than negative words, *p* < .001, 95% CI [5,35, 20.13], thus reflecting a general bias towards positive words [41], further analyses revealed that the main effect of encoding remained significant for both positively valenced recalled words, *F*(1,300) = 23.61, *p* < .001, η_p_^2^ = .11, and negatively valenced recalled words, *F*(1,300) = 31.05, *p* < .001, η_p_^2^ = .14, see Table 2. The simple effects mirrored the main effect comparisons.

### Discussion

Study 2 replicated the standard self-reference effect obtained in Study 1 using our novel online procedure. As hypothesized, participants recalled significantly more self-referentially encoded words than semantically encoded words and structurally encoded words, in both the liberal and the conservative data. Participants also recalled significantly more semantically encoded words than structurally encoded words. The self-reference effect therefore appeared stronger in Study 2 than in Study 1. We suggest that this was due to the slower presentation speed of the encoding questions, which encouraged more reflection than the participant-managed presentation speed used in Study 1. Alternatively, the effects may have been significant due to the increased statistical power afforded by the larger sample size of Study 2. The pattern of results in Study 2 is representative of the standard pattern of results obtained when comparing self-referential encoding to semantic encoding and structural encoding in the more traditional laboratory context [6].

We also note that the recall of semantically encoded words was lower in Study 2 than Study 1. This may be due to the speed of presentation and subsequent encoding time. In Study 1, the time between encoding and recall was effectively shorter as participants completed the questions at a faster pace. Encoding at a semantic level may be particularly sensitive to delays between encoding and recall, unlike self-referential encoding which can in fact benefit from such delays, as suggested by prior research [6]. Once again, positively valenced words were recalled more than negatively valenced words, regardless of type of encoding used.

## Study 3

Studies 1 and 2 demonstrated a reliable self-reference effect in a new online context. In order to further validate the procedure, Study 3 tested the effect of a typical experimental variation used in the laboratory context. Symons and Johnson (1997) found that the self-reference effect was diminished when participants were asked to *recognize* encoded words from a list rather than completing the typical free-recall task. Symons and Johnson suggest that this difference is due to the recognition process providing retrieval cues for semantic memory that are ineffective for self-referential encoding because the self already serves as its own retrieval cue system [6]. However, more recent research has demonstrated that self-referential encoding can still improve performance on recognition tasks [45]. Study 3 therefore compared the self-reference effect obtained using a standard recall task to that obtained using a word recognition task. We hypothesized that this would lead to an interaction effect resulting from the standard self-reference effect being replicated when participants performed the recall task but attenuated when they completed the recognition task.

### Method

#### Participants and design

Participants were 202 Amazon MTurk Workers (47% women, *M*_age_ = 37.30, *SD*_age_ = 12.90, range 18–70) paid USD$0.85 to take part in a 3 (encoding level: self-referential, semantic, structural) 3 X 2 (recall type: free recall, recognition) mixed design (the pay rate was increased for Study 3 due to the fact that the recognition condition took longer to complete). Participants who completed the previous studies were excluded from taking part in the present study. Recall type was a between-subjects variable and encoding type was a within-subjects variable. The dependent variable was the proportion of correct words recalled or identified as a function of encoding level, except for when reporting the between-subjects results, in which case the absolute number of words recalled was used as the dependent variable.

#### Procedure and materials

Participants completed the encoding task, which was identical to that described in Study 2. All participants completed the standard filler task after which they were randomly allocated to either the free recall or the recognition condition. Those in the free recall condition were given 120 seconds to complete the recall task, as in Studies 1 and 2. Participants in the recognition condition were presented with a selection of 60 individual words and were asked to indicate whether these were words they had seen previously in the encoding phase, or were new words (by indicating whether the words were ‘Old’ or ‘New’). The answer options were displayed below the word. The 30 original words were intermixed randomly with 30 new words, matched for length and valence, and again chosen from Anderson’s ‘Likeableness ratings of 555 personality-trait words [32], see S2 Appendix. Finally, participants were debriefed and paid.

#### Demographics

Participants indicated their age, gender and level of education. No attention check was included in this study.

### Results

The means and standard deviations for the main effect of encoding in all studies are displayed in Table 1.

#### Conservative analyses: Primacy and recency words excluded

Mauchley’s test indicated that the assumption of sphericity was violated (*W* = 0.82, *p* < .001), therefore degrees of freedom were corrected using Huynh-Feldt estimates of sphericity. As reported for Study 1 and Study 2, the dependent variable was the proportion of words correctly recalled at each level of encoding. However, when reporting the between-subjects results (recall versus recognition), the dependent variable was necessarily reported as the absolute number of recalled words. There was a significant main effect of recall task, *F*(1,200) = 7.60, *p* = .006, η_p_^2^ = .003, such that more correct words were identified in the recognition condition (*M* = 6.50, *SD* = 1.88, with a range of 0–9, and a median of 7) than were recalled in the recall condition (*M* = 1.16, *SD* = 1.20, with a range of 0–6, and a median of 1). There was also a significant main effect of encoding, *F*(2,400) = 96.21, *p* < .001, η_p_^2^ = .28. A linear mixed effects analysis revealed that participants correctly identified significantly more self-referentially encoded words (*M* = 46.50%, *SD* = 25.49) than semantically encoded words (*M* = 35.68%, *SD* = 23.20), *β* = -10.82, SE = 2.17, *p* < .001, and structurally encoded words (*M* = 14.35%, *SD* = 15.75), *β* = -32.15, SE = 2.17, *p* < .001. Participants also identified significantly more semantically encoded words than structurally encoded words, *β* = -21.33, SE = 2.17, *p* < .001.

These main effects were qualified by the hypothesized interaction between encoding level and recall task, *F*(2,400) = 9.82, *p* < .001, η_p_^2^ = .043. Planned contrasts revealed that in the recall condition only, results followed the standard self-reference pattern, such that participants recalled significantly more self-referentially encoded words (*M* = 51.73%, *SD* = 34.45) than semantically encoded words (M = 30.49%, SD = 30.69), *β* = -21.24, SE = 4.04, *p* < .001, and structurally encoded words (M = 10.78%, SD = 18.71), *β* = -40.95, SE = 4.04, *p* < .001. However, as hypothesized, the self-reference effect was eliminated when participants performed the recognition task. Here there was no significant difference between the number of correctly recognized self-referentially encoded words (*M* = 41.27%, *SD* = 9.07) and semantically encoded words (*M* = 40.77%, *SD* = 9.77), *β* = -0.60, SE = 1.40, *p* = .904. However, there were significantly fewer structurally encoded words recognized (*M* = 17.86%, *SD* = 11.21) than self-referentially encoded words, *β* = -23.52, SE = 1.40, *p* < .001, and semantically encoded words, *β* = -22.92, SE = 1.40, *p* < .001.

#### Liberal analyses: Primacy and recency words included

The effects remained similar using the liberal data. There was a significant main effect of recall task, *F*(1,200) = 5.32, *p* = .022, η_p_^2^ = .002, such that more correct words were identified in the recognition condition (*M* = 7.64, *SD* = 2.06, with a range of 0–10, and a median of 8) than in the recall condition (*M* = 1.51, *SD* = 1.33, with a range of 0–7, and a median of 1). There was also a significant main effect of encoding, *F*(2,400) = 100.87, *p* < .001, η_p_^2^ = .32, such that participants correctly identified significantly more self-referential words (*M* = 46.77%, *SD* = 20.41) than semantic words (*M* = 33.18%, *SD* = 18.03), *β* = -13.60, SE = 1.77, *p* < .001, and structural words (*M* = 17.57%, *SD* = 14.91), ), *β* = -29.20, SE = 1.77, *p* < .001. The interaction was again significant, *F*(2,400) = 6.87, *p* = .002, η_p_^2^ = .03.

However, there were slight changes to the pattern of simple effects. The simple effect of encoding was again significant in the recall condition, showing significantly higher recall of self-referentially encoded words (*M* = 50.12%, *SD* = 27.47) than semantically encoded words (*M* = 28.92%, *SD* = 23.61), *β* = -21.20, SE = 3.30, *p* < .001, and structurally encoded words, (*M* = 15.96%, *SD* = 18.34), *β* = -34.15, SE = 3.30, *p* < .001. There was also a significant, albeit weaker, simple effect of encoding in the recognition condition, showing significantly higher identification of self-referentially encoded words (*M* = 43.50%, *SD* = 8.19) than semantically encoded words (*M* = 37.35%, *SD* = 8.05), *β* = -6.14, SE = 1.25, *p* < .001, and structurally encoded words, (*M* = 19.15%, *SD* = 10.37), *β* = -24.34, SE = 1.25, *p* < .001.

Using the liberal data, the potential impact of age, gender and education levels were analyzed using a mixed multilevel model. Results revealed no main or interactive effects for age (*p*s > .717), gender (*p*s > .869), or education (*p*s > .683). We also checked whether the effect of encoding differed for positively and negatively valenced words. A paired *t*-test revealed that overall positive words were recalled significantly more than negative words, *p* < = .025, 95% CI [0.65, 9.50], thus reflecting a positive bias [41], and further analyses revealed that the main effect of encoding remained significant for positively valenced recalled words, *F*(1,306) = 37.21, *p* < .001, η_p_^2^ = .14, and negatively valenced recalled words, *F*(1,402) = 40.11, *p* < .001, η_p_^2^ = .14, see Table 2. The simple effects mirrored the main effect comparisons.

We also examined the impact of valence and encoding type on words that participants failed to recognize. A paired *t*-test revealed a non-significant effect of valence on non-recognized words, *p* = .725, 95% CI [-3.89, 2.71], but a significant effect of encoding, *F*(1,402) = 80.01, *p* < .001, η_p_^2^ = .171 on non-recognized words. Reversing the effects observed for recognized words, there were significantly more structurally encoded non-recognized words (*M* = 31.19%, *SD* = 36.09) than semantically encoded non-recognized words (*M* = 11.55%, *SD* = 18.05), *p* < .001, 95% CI [-14.72, 24.57], and more semantically encoded non-recognized words than self-referentially encoded non-recognized words (*M* = 5.77%, *SD* = 10.26), *p* = 0.02, 95% CI [0.85, 10.70]. These findings reinforce the patterns reported in our main analyses in showing that the most common words that participants encoded but failed to recognize were those that had been encoded at the most superficial (i.e. structural) level, and that the least common were words encoded in relation to the self.

Finally, we also looked at whether valence influenced the false recognition of incorrect words (i.e. identifying incorrectly a word as one which had been seen previously). Here a paired *t*-test examining the valence of incorrect recognitions also revealed a significant difference between positive and negative valence, *p* < .001, 95% CI [-32.80, -20.33], but this time in the opposite direction—with participants falsely recognizing more negative words (*M* = 34.32%, *SD* = 43.48) than positive words (*M* = 7.76%, *SD* = 18.54).

### Discussion

Study 3 replicated the self-reference effect using our novel online paradigm. As hypothesized, participants recalled significantly more self-referentially encoded words than semantically encoded words and structurally encoded words. Participants also recalled significantly more semantically encoded words than structurally encoded words. This pattern of results was representative of the standard pattern of results obtained when comparing self-referential encoding to semantic encoding and structural encoding in the more traditional laboratory context [6].

However, at a more fine-grained level of analysis, Study 3 also replicated the 18 recognition studies incorporated in Symons and Johnson’s (1997) meta-analysis. Specifically, and in line with Symons and Johnson’s proposed explanation, we found that after correcting for primacy and recency effects, retrieval cues present in the recognition condition enhanced the effects of semantic encoding to a similar mnemonic level as self-referential encoding, thus eliminating the standard difference between self-referential encoding and semantic encoding. Symons and Johnson [6] proposed that this effect results from the fact that semantic encoding benefits from retrieval cues inherent in a recognition format, whereas self-referential encoding has its own built-in retrieval cues—namely, the self. The maintenance of the self-reference effect in the liberal recognition data, however, could be seen to support more recent research indicating that encoding information in relation to the self can strengthen memory even within recognition contexts [45]. However, this could also be explained by differences in delivery format. Recent research has questioned the level of attention of crowd-sourced participants, MTurkers in particular [46], and it could be that this discrepant effect in the liberal data reflects these contextual differences in participant engagement. This is a possibility that could be further explored using the online self-referential encoding paradigm.

## Study 4

Study 3 replicated the findings of a specific experimental variation to the self-reference effect using our novel online paradigm. Study 4 tested the effects of another experimental variation by comparing the usual incidental recall procedure with an informed recall paradigm. According to Symons and Johnson’s (1997) meta-analysis, prior knowledge of recall results in explicit rehearsal effects that strengthen semantic and structural encoding levels, and therefore diminish the self-reference effect. On this basis, we hypothesized that there would be an interaction between encoding level and recall paradigm, with the standard self-reference effect being replicated when the recall task was unexpected, but eliminated when participants had previously been made aware that they would be asked to perform a recall task.

### Method

#### Participants and design

Participants were 201 Amazon MTurk Workers (48% women, *M*_age_ = 36.47, *SD*_age_ = 11.83, range 20–68) paid USD$0.85 to take part in a 3 (encoding level: self-referential, semantic, structural) 3 X 2 (recall paradigm: incidental, informed) mixed design. Participants who completed the previous studies were excluded from the study. Recall paradigm was a between-subjects variable and encoding level was a within-subjects variable. The dependent variable was the proportion of correct words recalled as a function of encoding level, except in the case of the between-subjects results, where the absolute number of recalled words was used as the dependent variable.

#### Procedure and materials

Participants completed the same encoding task as in Study 2 and were randomly assigned to the incidental or informed recall condition. The incidental condition was identical to that in Studies 1 and 2. Participants in the informed recall condition were told prior to the encoding phase that they would be asked to recall the words in the encoding task. Participants in this condition were informed that their performance in this recall test would not affect their payment for completing the study. In order to account for the inevitable unsupervised nature of MTurk participation, they were also asked not write down any of the words during the encoding stage in order to avoid invalidating results. All participants then completed the encoding task, followed by the filler task and the recall phase. Finally, participants were debriefed and paid.

#### Attention check and demographics

We embedded one attention check in the demographics presented at the end of the survey (“For this question, please just click the option ‘Very much’”). Seven participants failed this check. Excluding these seven participants from analyses did not substantively change the results, but their data were nevertheless excluded from further analysis. Participants also indicated their age, gender and level of education.

### Results

The means and standard deviations for the main effect of encoding in all studies are displayed in Table 1.

#### Conservative analyses: Primacy and recency words excluded

Mauchley’s test indicated that the assumption of sphericity was violated (*W* = 0.84, *p* < .001), therefore degrees of freedom were corrected using Huynh-Feldt estimates of sphericity. As reported for Studies 1 and 2, the dependent variable was the proportion of words correctly recalled at each level of encoding. However, as in Study 3, when reporting the between-subjects results (incidental versus informed recall), the dependent variable was necessarily reported as the absolute number of words recalled. There was a marginal main effect of recall condition, *F*(1,191) = 3.68, *p* = .056, η_p_^2^ = .002, such that participants recalled more words in the informed condition (*M* = 4.89, *SD* = 3.48, with a range of 0–22, and a median of 4) than in the incidental condition (*M* = 3.40, *SD* = 2.38, with a range of 0–12, and a median of 3). There was a significant main effect of encoding *F*(1,191) = 33.14, *p* < .001, η_p_^2^ = .13, such that participants recalled significantly more self-referentially encoded words (*M* = 43.83%, *SD* = 33.22) than semantically encoded words (*M* = 29.94%, *SD* = 28.56), *β* = -13.88, SE = 2.86, *p* < .001, and structurally encoded words (*M* = 16.90%, *SD* = 21.63), *β* = -26.92, SE = 2.86, *p* < .001. Participants also recalled significantly more semantically encoded words than structurally encoded words, *β* = -13.04, SE = 2.86, *p* < .001. The interaction between encoding level and recall type was not significant, *F*(2,382) = 1.38, *p* = .253 η_p_^2^ = .006.

#### Liberal analyses: Primacy and recency words included

The effects remained similar using the liberal data. The main effect of encoding remained significant, *F*(2,382) = 43.72, *p* < .001, η_p_^2^ = .165, as did the specific comparisons between self-referentially encoded words and semantically encoded words, *β* = -17.88, SE = 2.36, *p* < .001, and self-referentially encoded and structurally encoded words, *β* = -24.34, SE = 2.36, *p* < .001. There was, however, a non-significant main effect of recall condition, *F*(1,191) = 1.10, *p* = .296, η_p_^2^ = .001. The interaction between encoding level and recall type remained non-significant, *F*(2,382) = 0.83, *p* = .439 η_p_^2^ = .004.

Using the liberal data, the potential impact of age, gender and education levels was analyzed using a mixed multilevel model. Results revealed no main effect or interaction for age (*p*s > .120), gender (*p*s > .283), or education (*p*s > .350). We also checked whether the effect of encoding differed for positively and negatively valenced words. Although a paired *t*-test revealed that positive words were recalled significantly more than negative words, *p* < .001, 95% CI [7.70, 18.44], further analyses revealed that the main effect of encoding remained significant for positively valenced recalled words, F(2,382) = 27.32, *p* < .001, ηp^2^ = .11, and negatively valenced recalled words, F(2,382) = 13.19, *p* < .001, ηp^2^ = .05, see Table 2. The simple effects mirrored the main effect comparisons.

### Discussion

Study 4 replicated the self-reference effect using our novel online paradigm. As hypothesized, participants recalled significantly more self-referentially encoded words than semantically encoded words and structurally encoded words. This effect persisted even when participants had prior knowledge of the memory component of the study. This pattern runs counter to meta-analytic findings reported by Symons and Johnson (1997), in which an an informed recall condition was found to eliminate the usual self-reference effect. Symons and Johnson hypothesized that this was due to conscious rehearsal effects that boost the performance in semantic and structural encoding conditions but not in the self-reference condition (presumably because self-referential encoding is already functioning at ceiling level).

Our failure to replicate this discrepancy between informed and incidental recall tasks may be due to unique differences in online crowd-sourcing environments, such as MTurk. As mentioned, we explicitly instructed participants to avoid using memory enhancement strategies, such as writing the words down during the encoding phase. Such explicit instructions were necessary given that the experiment runs unsupervised on participants’ own computers, and as such would not be necessary when running lab-based studies. We speculate that it may be this difference that caused the discrepancy in findings. That is, participants in the online context were given instructions designed to avoid conscious rehearsal, which may have eradicated the expected effect of rehearsal strategies on non self-referential encoding.

## Meta-analysis

Having conducted four independent studies to examine the capacity of our online paradigm to reproduce the self-reference effect, we conducted a meta-analysis to establish the overall reliability and size of this effect. We performed this meta-analysis using both the liberal data that included primacy and recency words, and the conservative data that excluded primacy and recency words. The effect sizes and results of the meta-analyses across the studies are displayed in Table 3.

**Table 3:** Summary of effect sizes across the studies (Cohen’s d.).

	Study 1 SRE vs. SEM	Study 2 SRE vs. SEM	Study 3 SRE vs. SEM	Study 4 SRE vs. SEM	Meta-analyzed mean effect size SRE vs. SEM (Heterogeneity Q)	Meta-analyzed significance test z (95% CI)
**Sample size**	103	150	202	203		
**Conservative data**						
Main effect comparisons	.16	.71	.44	.45	0.45 (8.87*)	4.59*** (0.257 to 0.640)
Incidental recall comparisons	.16	.71	.65	.41	0.49 (11.85**)	4.34*** (0.269 to 0.713)
Recognition comparisons	-	-	.05	-	-	-
Informed recall comparisons	-	-	-	.50	-	-
**Liberal data**						
Main effect comparisons	.41	.87	.51	.71	0.63 (8.55*)	6.49*** (0.439 to 0.819)
Incidental recall comparisons	.41	.87	.83	.61	0.69 (8.59*)	7.05*** (0.496 to 0.879)
Recognition comparisons	-	-	.77	-	-	-
Informed recall comparisons	-	-	-	.85	-	-

*Notes*: Conservative data: excludes any words recalled that occurred in the first three positions or last three positions of the encoding list. Liberal data: includes all words recalled including those that occurred in the first three positions or last three positions of the encoding list.

SRE = self-referential encoding; SEM = semantic encoding.

* *p* < .05.

** *p* < .01.

*** *p* < .001

Effect sizes varied across studies. The effect size is lowest in Study 1, reflecting the lack of time participants spent on the encoding tasks due to there being no time constraint in the presentation of words in this study. From Study 2 onwards, all encoding questions were slowed down to ensure that participants took a standardised 5 seconds to read and answer each encoding question, and effects sizes increased as a result.

The mean weighted effect size (*d*) for self-referentially encoded words over semantically encoded words was 0.63 (0.45 with conservative analyses). Using only the data from the incidental free recall conditions (the standard self-reference procedure), the mean weighted effect size (*d*) for self-referentially encoded words over semantically encoded words was 0.69 (0.49 with conservative analyses). These effect sizes are comparable to the mean weighted effect size (*d*) of 0.65 reported in Symons and Johnson’s (1997) meta-analysis of 60 self-referential vs. semantic encoding studies. Nevertheless, the slight reduction in effect size in the conservative analyses could be due to qualitative differences between a laboratory sample and an MTurk sample. MTurk workers have been shown to be diligent participants [47], but as a workforce they are acutely aware of their fee per hour [48], and, as we saw with Study 1, this may mean that they adopt an expeditious orientation to the encoding task (wanting to get through it as quickly as possible) and that this then diminishes the encoding effects under observation. However, as we also saw from Study 2 onwards, it is possible to address (and manipulate) these factors through adjustments to the online procedure.

## General discussion

The self-reference effect has been used in a wide range of psychological research since it was first reported in 1977. With the emergence of neuroscientific research in the 1990s, the effect has taken on a new significance in deepening our understanding of how the self is represented neurally. With this resurgence of interest, it will be important to develop paradigms that are time and cost effective and that can reach a wider range of participants in studies that utilize large sample sizes, thus benefitting from more robust and replicable results [29–31]. In the present paper we have developed and tested one such paradigm in the form of an online version of the self-referential encoding task.

The results of the four studies reported here demonstrate the reliability the self-reference effect in this new online context. Participants recalled significantly more self-referentially encoded words than semantically encoded or structurally encoded words when the timing of the encoding task was unrestricted (Study 1) and restricted (Study 2). Study 3 also replicated an established boundary condition to the self-reference effect, such that a recognition task eliminated the effect relative to the usual recall task (Symons & Johnson, 1997). Study 4 again replicated the effect, but also highlighted one potential point of difference when administering this procedure online, as opposed to in a supervised laboratory context. Specifically, where an informed recall task eliminated the self-reference effect in a laboratory context, we found no such moderation in Study 4. This is most likely due to the fact that participants in the unsupervised online context had to be explicitly instructed to avoid rehearsal strategies, thus potentially eliminating the typical effects of strategies that are suggested to lead to an improvement in semantic and structural encoding relative to self-referential encoding. It is noteworthy too, that the overall effect size of self-referential encoding over semantic encoding within this online context is comparable to that previously reported in the meta-analysis of Symons and Johnson [6].

Results comparing the conservative and liberal data sets consistently show a stronger self-reference effect within the liberal data set that included primacy and recency effects (i.e., the first and last remembered three words). These differences in data sets could be explained by the simple reduction in number of encoding questions in the conservative data sets, although we would argue that this is unlikely given that only Study 1 reveals a non-significant self-reference effect in the conservative data set. Future studies using the online self-referential encoding tool would be able to investigate this question further.

We also analyzed the impact of positive and negative valence on encoding, and all four studies demonstrated that significantly more positive words were recalled than negative. Despite this difference, the typical self-reference effect persisted for both positive and negative words. The development of an online self-referential encoding paradigm will allow for much larger scale investigations into the possible impact of contextual effects on the interactions of encoding levels and valence.

## Future directions

Investigations using the self-reference effect provide a highly effective method with which to explore the self as it functions in a range of different contexts. Our studies laid out the foundations for a new reliable online self-referential encoding tool. Future studies can build on these foundations, and statistical methodologies such as Signal Detection Theory, will be particularly important when it comes to investigating contextual difference in self-referential encoding. For example, we did not investigate reaction time, which, along with valence, is a useful indicator of automaticity of response and self-schema availability. Further investigations into the particular influence of valence would be highly beneficial for research in the clinical domain. Furthermore, our studies did not investigate time variation between encoding and recall, which could potentially shed further light on the dual nature of self-referential encoding in which both elaborate and organisational encoding may prove more influential at different durations [49].

Looking further ahead, an area that would benefit from the accessibility and scalability of the online self-referential encoding tool is developmental research. We did not observe any significant effect of age on encoding levels (presumably due to negative skew towards younger adults, *Mean* = 32.26, *Median* = 28.75). However, studies included in Symons and Johnson’s meta-analysis [6] investigated differences in levels of encoding between children and adults, and their results demonstrated significantly higher levels of self-referential encoding for adults [50, 51]. A more recent study by Cunningham, Brebner, Quinn and Turk [52] also investigated the self-reference effect in early childhood. With the availability of an online tool to assess levels of self-referential encoding, these developmental investigations will be able to expand exponentially: allowing researchers to explore theoretical underpinnings of the developmental pathway that gives rise to the superiority of the self as a cognitive schema.

A further area of psychological research that could benefit from the online capability of the self-reference paradigm concerns investigation into the way in which cultural orientation can alter basic cognitive, emotional and behavioral processes [53, 54]. The self-reference paradigm has been central to many of these recent studies. For example, research by Zhou, Zhang, Fan and Han [17] used this paradigm to explore the difference between self- and other-referential processing for Western and Chinese participants, and demonstrated a significant distinction between self and intimate other-referential encoding for Western participants that was not evident for Chinese participants. Research by Choi, Kang and Sul [15] also investigated different types of self-referential encoding—specifically comparing personal traits versus social identities, and demonstrated that individualistic cultural orientation was associated with higher levels of self-referential encoding for personality traits, whereas collectivist cultural orientation was associated with higher levels of social identity-related encoding. These studies used a laboratory version of the self-referential encoding paradigm. With the availability of a reliable online version, studies to explore the impact of cultural orientation on self-related cognitive processing can recruit participants from farther afield and with greater statistical power [29–31].

Another area of research that would benefit from the availability of the online self-referential encoding tool is the study of psychological boundaries between self and other [55, 56]. Bower and Gilligan [28] observed that encoding information in relation to a significant other (e.g., “Does the word describe your Mother?”) can result in memory traces as strong as those for self-referentially encoded material. Along these lines, research by Aron, Aron, Tudor and Nelson [57] investigated processing differences between Self, Mother and Stranger—observing that the processing of Mother was more akin to the processing of Self rather than of Stranger. Symons and Johnson (1997) point out that the level of intimacy with the target ‘other’ determines the relative power of the other-reference effect and diminution of the self-reference effect. This suggests that other- and self-reference effects could in fact be used to measure how, when, and to what degree the other becomes internalized within the self (e.g., in ways suggested by self-categorization theory; Turner & Oakes, 1989 [58]; Turner, Oakes, Haslam, & McGarty, 1994 [59]). Investigating the intricacies of these processes for different populations and within different contexts becomes not only more feasible through the use of an online version of the self-reference paradigm, but also more statistically powerful.

## Limitations

As with all research, the present studies had a number of limitations that might be addressed in future work. For example, two words in the lists were displayed at a smaller font size than the others to fit on the screen. Future work might replace these with shorter words to confirm that their inclusion did not influence the results (although we note this is unlikely due to randomization of encoding level across the word lists).

Words were matched on positive and negative valence, as well as being matched on yes/no response when delivered with either a semantic or a structural encoding question (see the Procedures section of Study 1 for more details). However, words were not matched on frequency or arousal. Research within the field of encoding has shown that these factors have the potential to impact on endorsement and recall [2]. Finally, in future work it will be important to ensure that participants have a basic level of proficiency with the English language to ensure effective participation in the studies.

## Conclusions

The online self-reference paradigm provides a reliable procedure with which to measure self-referential encoding in a variety of different contexts and with a wide range of populations. Testament to this, the average data sample size for these four studies was 165 participants as opposed to an average of 39 for the 126 studies included in Symons and Johnson’s (1997) meta-analysis [6]. Of those same 126 studies, 82% were drawn from undergraduate populations. In contrast, the 658 participants recruited for these four online studies had a much more diverse profile. Across the four studies 47% of participants were women, with a mean age of 36.63 (*SD*_age_ = 12.44, range 18–71). Their maximum level of education was also varied: 14% had completed high school, 24% had an incomplete bachelor’s degree, 37% had bachelor’s degree, 1% had a PhD, 12% had a graduate or professional degree, and 12% had an associate degree. This new online procedure therefore extends the accessibility, power and scope of investigations into the self-reference effect and possibilities for investigating the self more generally. Quickly and easily administered, the online self-reference paradigm can be deployed wherever there is online access, and can be used to collect data from samples of unprecedented size that far exceed the power of studies administered in a traditional laboratory context.

## Supporting information

S1 Appendix R files. All files are available in S1.
(ZIP)

S2 Appendix Word lists. Sample word lists.
(PDF)

S3 Appendix Encoding paradigm. Sample encoding paradigm screens plus online link to encoding paradigm demonstration.
(PDF)
