# Supplementary material for: An online paradigm for exploring the self-reference effect
Source: PLoS One. 2017 May 4;12(5):e0176611. doi: 10.1371/journal.pone.0176611 (PMC5417556; doi:10.1371/journal.pone.0176611)
Supplement: S2 Appendix — Sample word lists. (PDF) [file pone.0176611.s002.pdf]

Appendix: Word lists

Study 1 Word List

absent-minded*	foolish	reliable
adventurous	friendly	resourceful
average	helpless	selfish
calm	insincere	smug
conventional	neat	tactful
courageous	neglectful	tense
cowardly	observant	thoughtful
deceitful	optimistic	tough
dominating	practical	untrustworthy*
experienced	relaxed	wise

* Words presented at the reduced size of 48 pt due to their length.

Study 2 Word List

adventurous	foolish	reliable
average	friendly	resourceful
calm	helpless	selfish
conventional	immature	smug
courageous	neat	tactful
cowardly	neglectful	tense
deceitful	observant	thoughtful
discourteous*	optimistic	tough
dominating	practical	untrustworthy*
experienced	relaxed	wise

* Words presented at the reduced size of 48 pt due to their length.

Study 3 Recognition Word List

'Old' words	'New' words
adventurous	understanding
average	skeptical
calm	nice
conventional	undecided
courageous	proficient
cowardly	resentful
deceitful	envious
discourteous	antisocial
dominating	unproductive
experienced	inquisitive
foolish	choosy
friendly	pleasant
helpless	unlucky
immature	eccentric
neat	able
neglectful	rebellious
observant	studious
optimistic	enterprising
practical	thorough
relaxed	decisive
reliable	popular
resourceful	vigorous
selfish	scheming
smug	rude
tactful	efficient
tense	weak
thoughtful	refined
tough	direct
untrustworthy	inattentive
wise	warm
