# Supplementary material for: An online paradigm for exploring the self-reference effect
Source: PLoS One. 2017 May 4;12(5):e0176611. doi: 10.1371/journal.pone.0176611 (PMC5417556; doi:10.1371/journal.pone.0176611)
Supplement: S3 Appendix — Sample encoding paradigm screens plus online link to encoding paradigm demonstration. (PDF) [file pone.0176611.s003.pdf]

# Appendix: Encoding paradigm

## Sample Encoding Screens

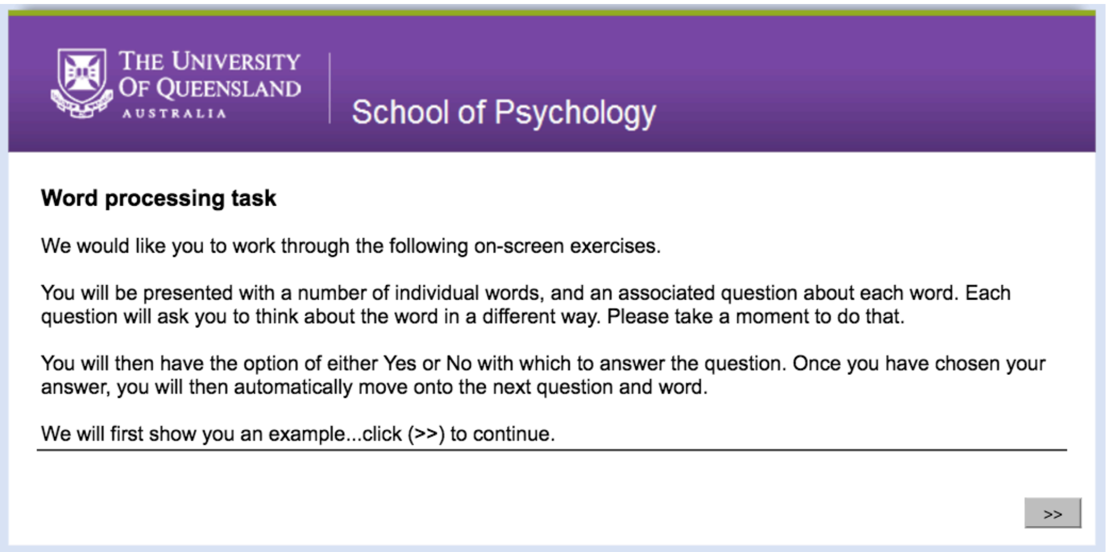


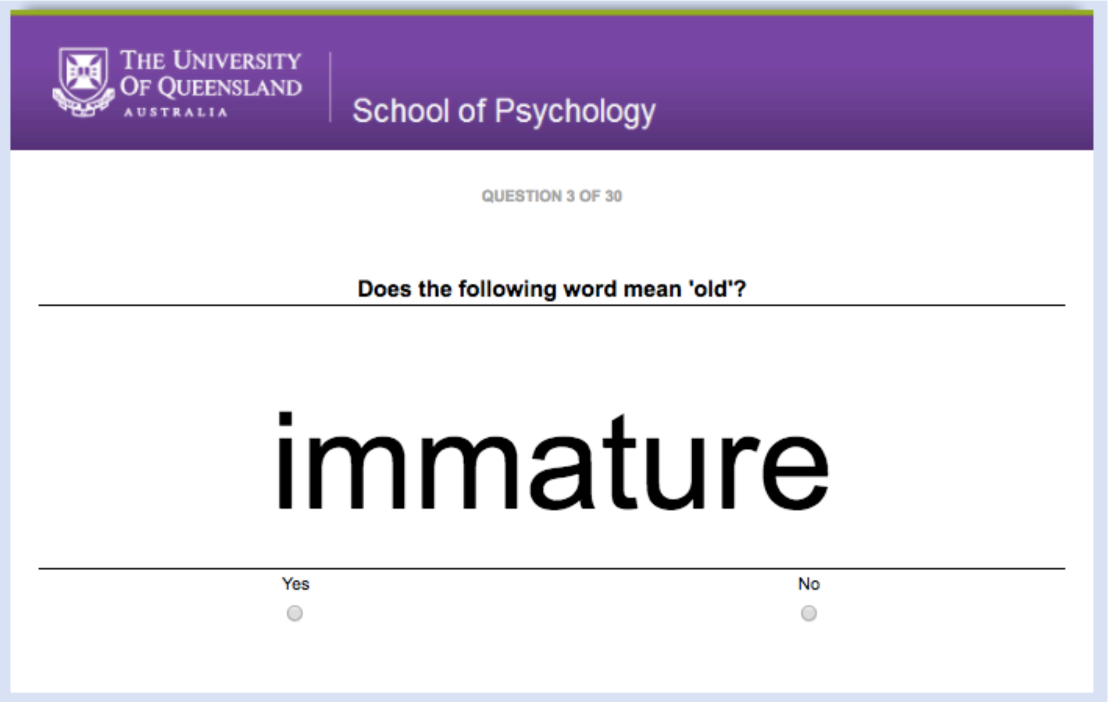

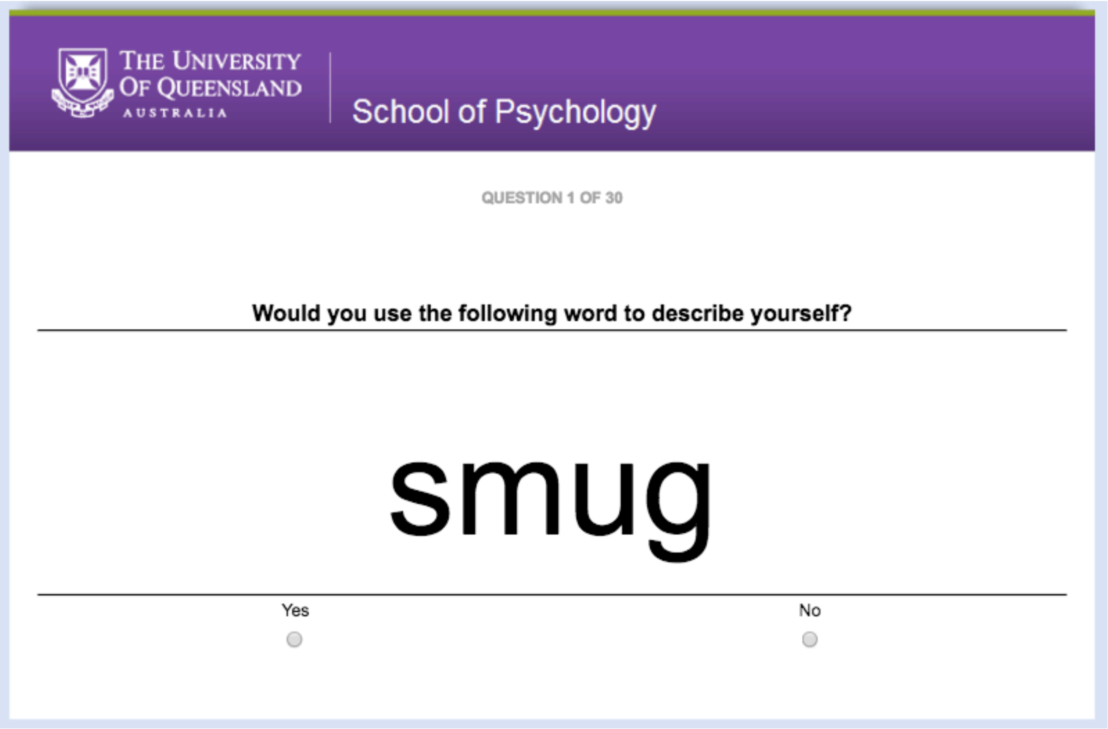
The University
Of Queensland
Australia
School of Psychology
QUESTION 1 OF 30
Would you use the following word to describe yourself?
smug
Yes
No

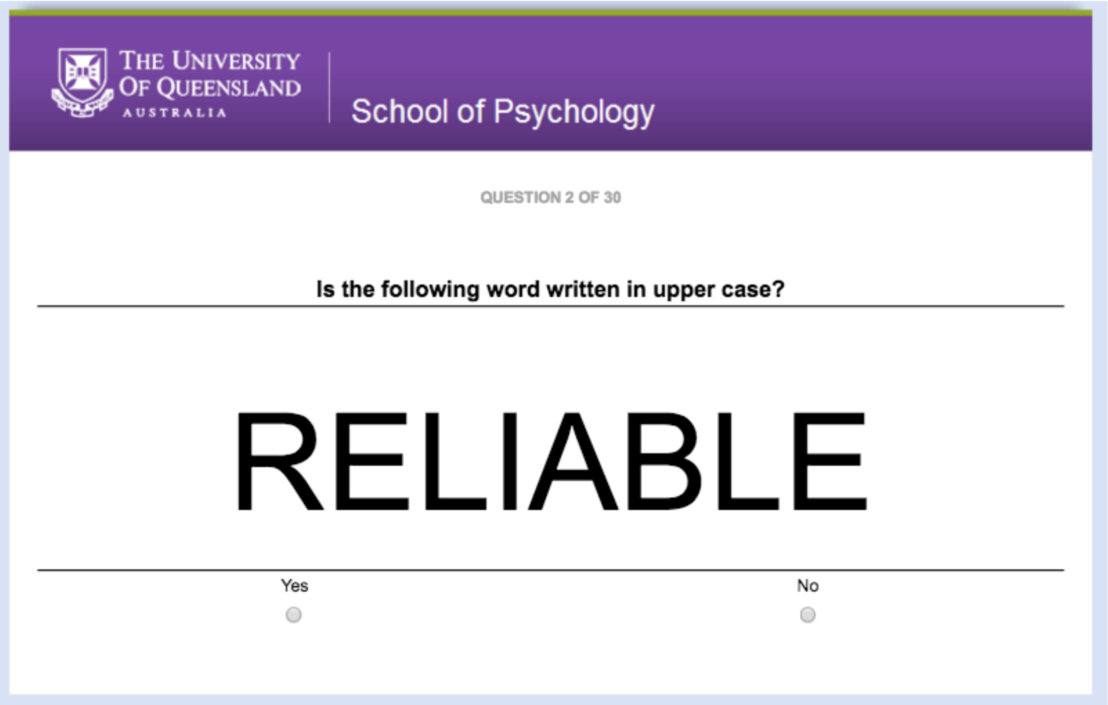
The University
Of Queensland
Australia
School of Psychology
QUESTION 2 OF 30
Is the following word written in upper case?
RELIABLE
Yes
No

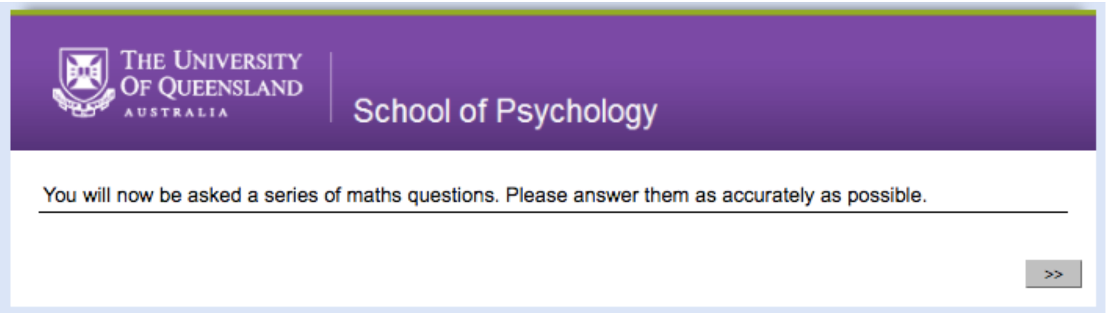
The University of Queensland
Australia
School of Psychology
You will now be asked a series of maths questions. Please answer them as accurately as possible.
>>

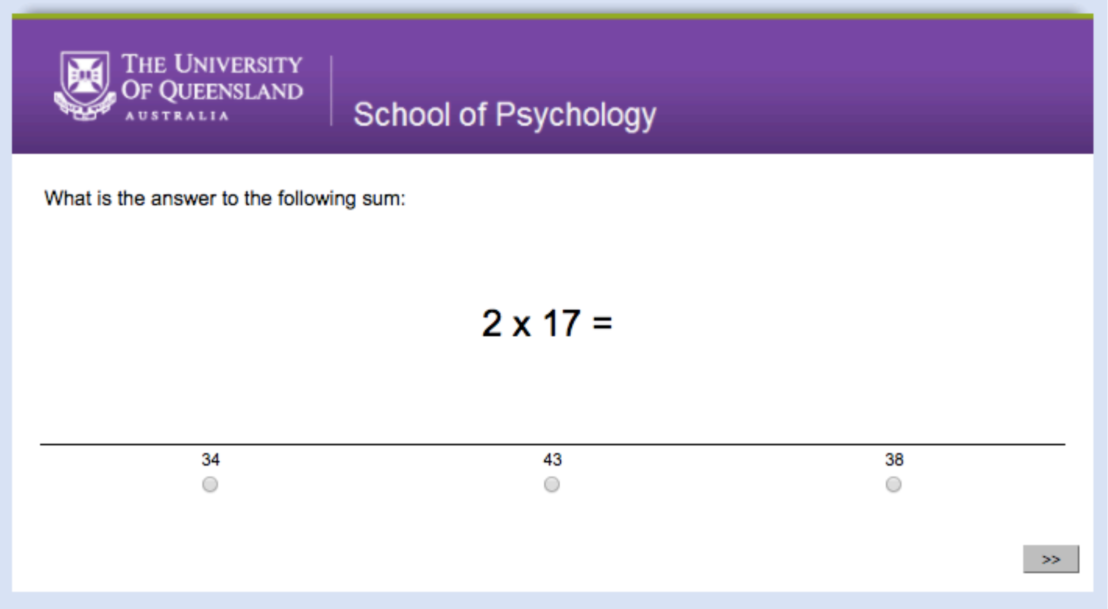
The University of Queensland
Australia
School of Psychology
What is the answer to the following sum:
2 x 17 =
34
43
38
>>

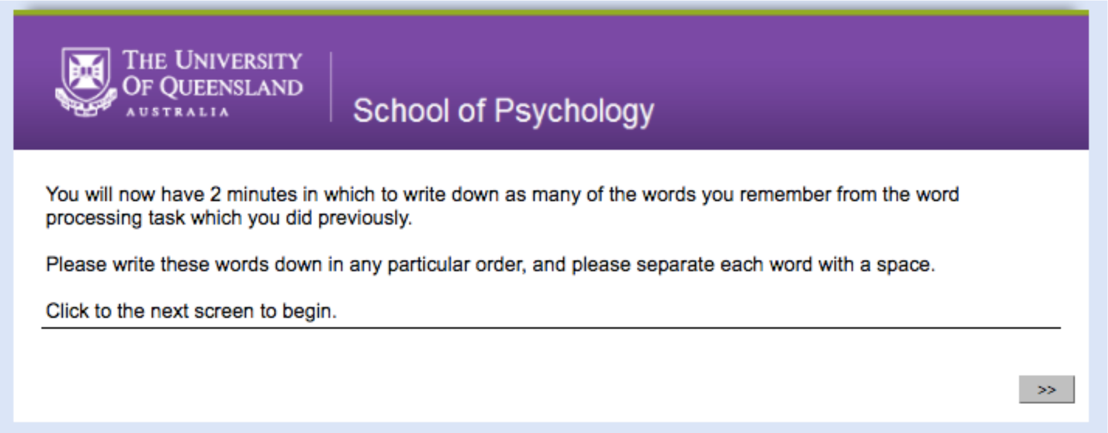
The University of Queensland
Australia
School of Psychology
You will now have 2 minutes in which to write down as many of the words you remember from the word processing task which you did previously.
Please write these words down in any particular order, and please separate each word with a space.
Click to the next screen to begin.
>>

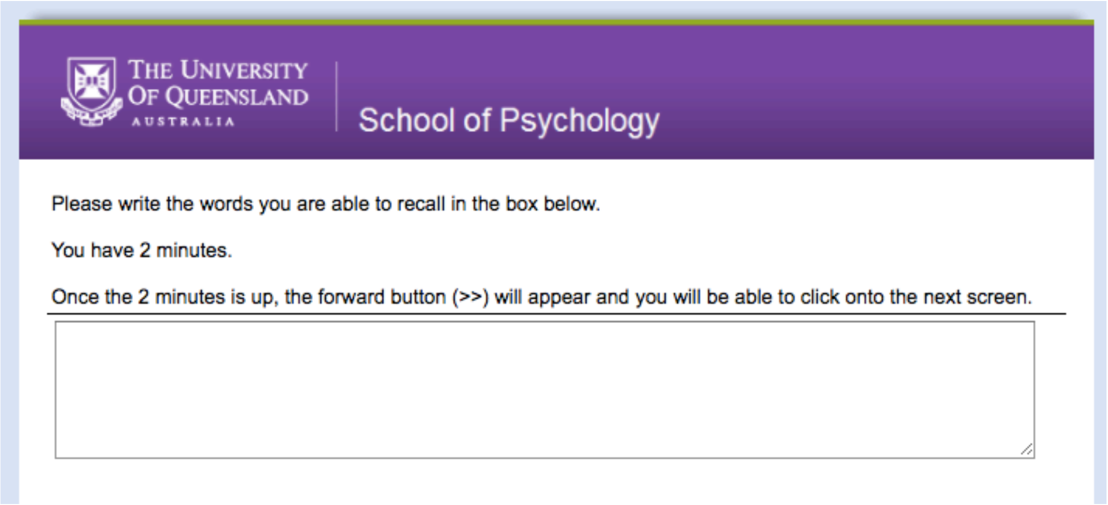


A full demonstration of the on-line self-referential encoding paradigm can be accessed here: tinyurl.com/Bentley-Sample-SRE-online.
